# Genetic, Environmental and Lifestyle Determinants of Accelerated Telomere Attrition as Contributors to Risk and Severity of Multiple Sclerosis

**DOI:** 10.3390/biom11101510

**Published:** 2021-10-13

**Authors:** Michael Hecker, Jan Bühring, Brit Fitzner, Paulus Stefan Rommer, Uwe Klaus Zettl

**Affiliations:** 1 Division of Neuroimmunology, Department of Neurology, Rostock University Medical Center, Gehlsheimer Str. 20, 18147 Rostock, Germany; jan.buehring@uni-rostock.de (J.B.); brit.fitzner@uni-rostock.de (B.F.); paulus.rommer@meduniwien.ac.at (P.S.R.); uwe.zettl@med.uni-rostock.de (U.K.Z.); 2 Department of Neurology, Medical University of Vienna, Währinger Gürtel 18–20, 1090 Vienna, Austria

**Keywords:** multiple sclerosis, telomere length, telomere shortening, aging, genetics, environment, lifestyle, risk factors, disease modifiers

## Abstract

Telomeres are protective structures at the ends of linear chromosomes. Shortened telomere lengths (TL) are an indicator of premature biological aging and have been associated with a wide spectrum of disorders, including multiple sclerosis (MS). MS is a chronic inflammatory, demyelinating and neurodegenerative disease of the central nervous system. The exact cause of MS is still unclear. Here, we provide an overview of genetic, environmental and lifestyle factors that have been described to influence TL and to contribute to susceptibility to MS and possibly disease severity. We show that several early-life factors are linked to both reduced TL and higher risk of MS, e.g., adolescent obesity, lack of physical activity, smoking and vitamin D deficiency. This suggests that the mechanisms underlying the disease are connected to cellular aging and senescence promoted by increased inflammation and oxidative stress. Additional prospective research is needed to clearly define the extent to which lifestyle changes can slow down disease progression and prevent accelerated telomere loss in individual patients. It is also important to further elucidate the interactions between shared determinants of TL and MS. In future, cell type-specific studies and advanced TL measurement methods could help to better understand how telomeres may be causally involved in disease processes and to uncover novel opportunities for improved biomarkers and therapeutic interventions in MS.

## 1. Telomere Biology

In mammals, the end structure of every chromosome arm is composed of a repetitive tandem DNA sequence of the hexanucleotide TTAGGG and a 50–400 nucleotide long single-stranded 3′ overhang. Human telomeres are typically between 5 and 15 kilobases (kb) long [1,2]. Shelterin protein complexes are part of the telomere structures and aid in protecting the chromosome ends from DNA damage response pathways [1,3]. During the physiological aging process, the telomere length (TL) continuously decreases because of a variety of reasons. One reason is the so-called end-replication problem, which results in a loss of ~50–100 base pairs (bp) per cell division [1,4]. Hence, for normal cells, there is a limit of replicative capacity, which is called the Hayflick limit [5]. Critically short telomeres lead to cellular senescence and eventually cell death. Apart from the age-related shortening of telomeres, numerous genetic and nongenetic factors can affect the rate of telomere attrition, as we will discuss later in this paper.

Telomere shortening with age can be accelerated by enhanced systemic oxidative stress (OS) and chronic inflammation, which provokes increased cellular turnover. OS is defined as an imbalance between the production of reactive oxygen species (e.g., by mitochondria) and the elimination of said species by antioxidants. Telomeres are susceptible to damage by OS because of their guanine-rich structure, and faster telomere shortening due to OS has been demonstrated in vivo and in vitro [6,7]. For the indirect measurement of OS, the resulting damage to DNA and proteins can be determined. For instance, elevated levels of 8-hydroxydeoxyguanosine (8-OHdG, a marker for oxidative DNA damage) and protein carbonyl (oxidation products of proteins) are associated with shorter TL [8]. A correlation coefficient of *ρ* = −0.641 between 8-OHdG concentrations and leukocyte telomere length (LTL) was reported for patients with type 2 diabetes mellitus [9]. Plasma levels of acute-phase proteins such as C-reactive protein (CRP) and serum amyloid A (SAA) and of other indicators of inflammation such as TNF-α and IL-6 have also been associated with shorter LTL [8,10]. Moreover, chitinase 3-like protein levels were found to correlate negatively with spleen TL in mice (*r* = −0.636) [11] and with LTL in humans (*r* = −0.547) [12]. Chitinase enzyme activity in blood plasma is a biomarker of human aging and is elevated in aging-associated and inflammatory diseases [13].

Telomere lengthening can counteract the loss of terminal DNA sequences. Telomeres can be elongated by a ribonucleoprotein enzyme complex containing telomerase reverse transcriptase (TERT) and a functional ribonucleic acid (RNA) called telomerase RNA component (TERC) [14]. TERC serves as a template to synthesize telomeric repeats at the chromosomal ends. Most human cells express low levels of telomerase, with the exception of germ line cells and embryonic stem cells, where physiologically higher concentrations can be measured [15]. TERT transcription is influenced by several determinants, such as hormones and viral infections [1]. The expression of TERT is also controlled by the length of telomeres via a feedback mechanism [16]. In cancer cells, telomerase activity is often highly up-regulated, which correlates with the capacity for unlimited cell proliferation [17]. However, in ~10–15% of human cancers, a telomerase-independent recombination-mediated mechanism called alternative lengthening of telomeres (ALT) is utilized [18].

For measuring TL, several techniques have been invented within the last years. The Southern blot analysis of terminal restriction fragments (TRF) is often denoted as the “gold standard” method. Real-time quantitative polymerase chain reaction (PCR) assays to determine relative TL were developed by Cawthon [19,20]. In these assays, telomere signals (T) and single-copy gene signals (S) are measured and used to calculate relative T/S ratios, which are proportional to the average TL. Samples that show a T/S ratio >1.0 contain longer telomeres than the reference DNA sample used. For analyzing single telomeres and the abundance of the shortest telomeres, improved methods such as quantitative fluorescence in situ hybridization (Q-FISH), universal single telomere length analysis (U-STELA) and telomere shortest length assay (TeSLA) are suitable [21,22]. While Q-FISH is based on the specific labeling of telomeric repeats with fluorescent markers, U-STELA and TeSLA are based on adapter ligation and PCR combined with Southern blot. However, as reviewed elsewhere [22], all these methods have some limitations. For instance, the metaphase Q-FISH method requires proliferating cells, and U-STELA is not efficient for determining telomeres greater than 8 kb. New approaches for TL analysis based on digital PCR [23] and long-read sequencing [24] have been proposed recently.

Much research has been dedicated to understanding the association between telomere biology and disease. Relatively short TL as indicator of premature biological aging has been observed in various chronic neurological and inflammatory disorders [25,26]. In patients with cancer, both excessively short and long TLs have been demonstrated, dependent on tumor type [27]. Four recent studies provided evidence that LTLs are shorter in patients with multiple sclerosis (MS) than in healthy controls (standardized mean difference (SMD) = −0.66) [28,29,30,31,32]. Shorter telomeres in MS patients were also found to be associated with greater disability and brain atrophy as well as disease progression independent of chronological age, as we have discussed in a previous review [32]. Recent data from the UK Biobank indicated that longer telomeres are related to a higher risk of developing MS later in life (adjusted hazard ratio (HR) per standard deviation (SD) longer LTL = 1.27) [33]. This somewhat contradictory finding may suggest that shortened TLs in MS patients result from a higher rate of telomere attrition and/or as a consequence of pathophysiological processes. In most studies, leukocyte TL has been analyzed because of the ease of obtaining whole blood samples. TLs are generally positively correlated among different types of tissue [34]. Nevertheless, a better understanding of TL variation across human tissues and cell types may help to further delineate the relationship with age and the role of telomere-mediated mechanisms in disease risk.

## 2. Multiple Sclerosis

MS is a chronic immune-mediated neurodegenerative disease of the central nervous system (CNS) and the primary cause of nontraumatic disability in young adults [35]. About 2.8 million people live with MS worldwide [36]. The exact cause of MS is still unknown, but it is generally accepted that the disease is the result of complex gene–environment interactions [37]. The clinical presentation of individual MS patients is very heterogeneous because of the variable location and number of demyelinating lesions that occur in the brain and/or spinal cord. Disease symptoms range from relatively slight to very severe and include motor impairments, visual loss due to optic neuritis, sensory deficits, fatigue and slowed cognitive processing [38,39]. The disease is typically diagnosed at between 20 and 40 years of age, and females have a roughly three times higher risk to develop MS than males [40,41].

MS is characterized by the occurrence of relapses (episodes of new or worsening neurological symptoms) and progression of cognitive and physical disability. The patients’ degree of disability can be rated with the Expanded Disability Status Scale (EDSS), which ranges from 0 (normal neurologic exam) to 10 (death as a result of MS) [42]. The diagnostic criteria for MS are based mainly on the clinical presentation (e.g., relapse occurrence) and on lesion activity as assessed using magnetic resonance imaging (MRI) [43]. To exclude alternative diagnoses and to affirm intrathecal inflammatory processes, the laboratory analysis of cerebrospinal fluid can be useful [44]. Different clinical courses of MS can be distinguished [45]: relapsing–remitting MS (RRMS), secondary progressive MS (SPMS) and primary progressive MS (PPMS). The first clinical event that is suggestive of MS is called clinically isolated syndrome (CIS). Approximately 85–90% of the patients are initially diagnosed with RRMS, while 10–15% present with PPMS [46,47]. RRMS is characterized by the occurrence of acute relapses followed by periods of partial or complete remission, whereas patients with PPMS show progressive worsening of disability from onset [45]. Within a disease duration of ~20 years, roughly half of the patients with RRMS develop SPMS, a phase of irreversible disease progression that is independent of the presence of relapses [47].

The first choice for the treatment of acute relapses is glucocorticoids. In the case of steroid-refractory relapses, an escalating therapy with apheresis may be indicated [48,49]. In the past years, a wide range of disease-modifying treatments for MS have been approved to prevent new lesions, reduce the frequency of relapses and slow down the rate of disability worsening [50]. These treatments have substantially improved the outlook for many patients. They act by suppressing or modulating immune functions, depleting (autoreactive) lymphocytes and/or inhibiting their migration into the CNS, as reviewed elsewhere [51]. However, undesirable side effects may occur during the course of treatment [52], and long-term prognosis of disease activity for individual patients remains difficult [53,54].

Our understanding of the immunopathology of MS has constantly improved over the years, although the precise molecular mechanisms that contribute to disease development and progression remain to be elucidated. The disease is thought to be mediated by an interplay of circulating immune cells (such as T and B cells) and CNS-resident cells (such as microglia and astrocytes) [55]. Key steps in the pathogenesis of MS are presumably the peripheral expansion of autoreactive immune cells, the breakdown of the blood–brain barrier [56], the infiltration of activated cells into the CNS and the subsequent generation of inflammatory lesions that are associated with myelin sheath destruction and progressive axonal loss [35]. Within the CNS, T cells are reactivated by local antigen-presenting cells. This induces the recruitment of additional leukocytes [57]. The crucial role of CD4^+^ and CD8^+^ T cell responses in the pathogenesis of MS is further underlined by the strong association of genes encoding human leukocyte antigen (HLA) class I and II molecules, which present antigenic peptides to T cell receptors, with MS susceptibility, which is described in more detail in the following section. B cells are also tightly linked to MS as they present antigens, produce cytokines and differentiate into antibody-secreting cells [58,59,60]. Activated B cells from individuals with MS produce abnormally high amounts of the cytokines TNF-α and IL-6, the latter of which is particularly involved in the generation of Th17 cell responses [61]. B cells of MS patients also express increased levels of costimulatory molecules (such as CD80 and CD86), rendering them highly potent activators of autoreactive T cells [62]. Senescent cells may promote the inflammatory and demyelinating processes in MS by secreting high levels of proinflammatory cytokines [63]. As oligodendrocytes are damaged over time, iron is released, and its extracellular accumulation potentiates OS and results in further neurodegeneration [64].

Aging has profound effects on the pathobiology and course of MS [65]. Aging is related to immunosenescence, a weakened capacity for mounting adequate immune responses, and it is often accompanied by inflamm-aging, a chronic age-related increase in the levels of proinflammatory markers in blood and tissues. Initiation and progression of MS and the transition from an inflammatory to a neurodegenerative disease phenotype are presumably linked to such age-dependent immunological and neuropathological changes [66]. More specifically, telomere attrition, genomic instability and cellular senescence may disturb neuro–immune interactions in MS and may impair effective debris clearance, remyelination and brain injury repair. However, the relative contributions of age-related processes to clinical deficits remain largely unclear, which complicates disease management [67]. Interestingly, several molecular biomarkers of MS also correlate with TL as a measure of the biological age. In the blood of patients with MS, elevated levels of markers for OS, e.g., 8-OHdG and protein carbonyl, and a lower antioxidant capacity have been reported [28,29,68,69,70,71]. However, it is unclear whether elevated OS markers are a cause or a consequence of MS. Signs of systemic inflammation, as reflected, for instance, by moderately increased serum levels of SAA and CRP, were also detected in untreated MS patients compared to healthy controls [72,73]. Telomere shortening is also related to higher levels of chitinase 3-like protein 1 (CHI3L1), which were found in cerebrospinal fluid and serum at different disease stages and associated with a higher conversion rate from CIS to RRMS [74]. Moreover, CHI3L1 levels correlated with higher EDSS scores in patients with PPMS (*ρ* = 0.490) [75].

**Figure 1:**
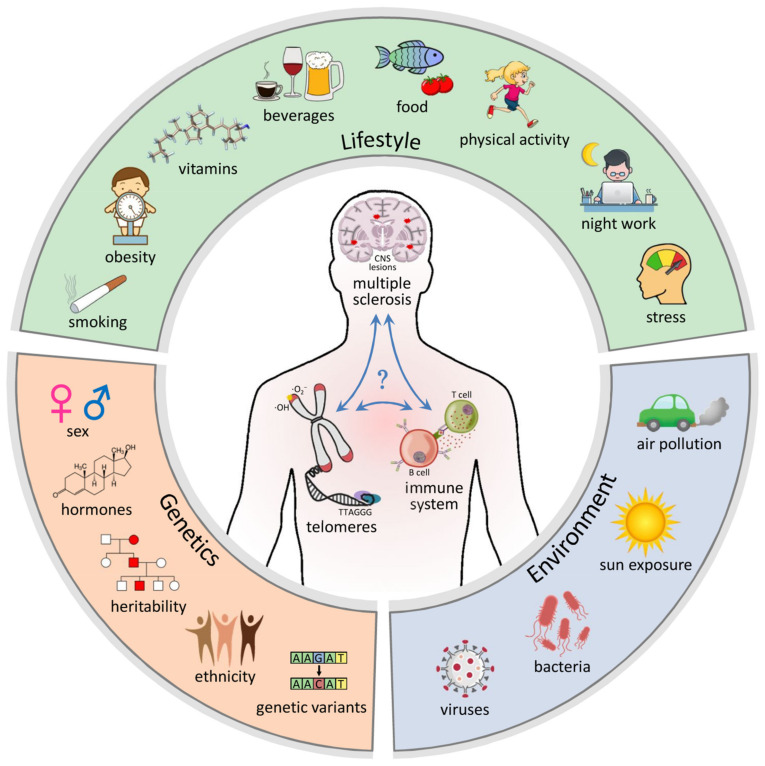
Common factors known to influence telomere length as well as susceptibility and course of multiple sclerosis (MS). A combination of genetic, environmental and lifestyle/behavioral factors contributes to risk and severity of MS. Shown in the outer ring are MS risk factors that have also been described to influence the shortening of telomere lengths (TL) with age. Accelerated loss of telomeric repeats, mediated by increased oxidative stress and chronic inflammation, can lead to genomic instability and altered immune cell functions. In MS, this may contribute to an abnormal response of the immune system that is directed against the central nervous system (CNS). Moreover, senescence mechanisms in CNS-specific cells may also play a role in the pathogenesis and progression of MS. It remains to be elucidated in more detail how exactly interactions between genetic and nongenetic factors may decisively shape the risk of MS in childhood/adolescence and the course of the disease in later life.

A better understanding of the onset and course of MS might be achieved by further exploring the complex interplay between pathologic immune responses, age-related telomere dynamics and immunosenescence. In the following subsections, we present an overview of genetic, environmental and lifestyle factors that have been described to be associated with risk and possibly severity of MS, on the one hand, and with TL, on the other hand. We considered lifestyle factors to be those that can usually be modified by the individual person with some effort. However, a clear distinction between modifiable and nonmodifiable factors is sometimes difficult. As a result, we found a number of shared risk factors that were described to influence both telomere biology and disease development (Figure 1 and Table 1). We discuss possible links between telomeres and the molecular mechanisms of MS. These insights may have implications for future patient care and may open new avenues for research on improved biomarkers and treatments.

## 3. Genetic Factors

### 3.1. Race/Ethnicity

Incidence and prevalence of MS increase with latitude, both north and south of the equator. Accordingly, the disease is more common in Europe, North America and Australia, while it is rare in Africa and Asia [36,76]. The estimated prevalence in Europe per 100,000 population is 142.8. The prevalence is even higher in the United States and Canada, with 288 and 250 cases per 100,000, respectively. In Asia, the lowest prevalence rates were found in China and Japan, with 3 and 4 cases per 100,000, respectively [36]. Apart from genetic risk profiles, other global differences may also explain the worldwide distribution of MS, e.g., sun exposure, spread of infectious agents and socioeconomic conditions. Nevertheless, genetic ancestry most likely explains a large part of the geographical variation in MS risk. Notably, a study focused on the east London area reported that the local prevalence of MS was 180, 74 and 29 for the White, Black and Asian populations, respectively [77]. In a province in South Africa, a much higher prevalence of MS in White people (25.6 in 100,000) than in Black people (0.2 in 100,000) was observed [78]. In contrast, Blacks had similar incidence rates compared to Whites (males: 8.4 vs. 7.3, females: 26.3 vs. 25.8) in a nationwide cohort from the US military-veteran population [79], suggesting that gene–environment interactions play a significant role in the etiology of MS. Inconsistent findings have been published with regard to disease outcomes: Some authors reported that African Americans with MS have a more severe disease course than Caucasian Americans [80], whereas others found that disability progression did not differ among racial/ethnic groups when access to health care and cultural views of illness were considered [81].

Differences in TL among racial/ethnic groups have been described. A recent study from the GTEx consortium showed that variation in TL is partly explained by genetic ancestry: On average, individuals of African ancestry had significantly longer relative TL than those of European ancestry across different adult tissues, e.g., cerebellum (1.19 vs. 1.02) and blood (0.90 vs. 0.81) [34]. This confirmed previous studies that found the mean LTL to be significantly longer by 0.3–0.6 kb in Africans than in Europeans [82,83]. The differences in LTL when comparing Blacks and Chinese to Whites were found to correspond to a difference in biological age of 17.9 and 15.6 years, respectively [84]. Interestingly, this gap is presumably related to the rate of telomere shortening from birth onward, as no significant LTL differences between ethnic groups were found in newborns [85]. The specific factors that define TL dynamics during the formative years and that might be associated to chronic disease later in life remain to be explored in depth.

### 3.2. Sex Differences

Sex differences have been shown for MS susceptibility, symptomatology and disease progression [86,87]. Overall, women have up to three times higher risk of developing MS than men [35,41], but there is no female preponderance among PPMS patients [88,89]. Hence, male patients more commonly present a progressive course of disease with more rapid accumulation of disability [90,91]. Brain atrophy and cognitive decline are also stronger in men in comparison to women affected by MS [92,93]. In contrast, female patients were reported to have an approximately 20% higher relapse rate [88].

No sex-specific differences in TL were found in studies with focus on MS [32]. However, other studies showed that there is little difference in TL between the sexes at birth [85], but thereafter, males tend to have shorter telomeres than females, indicating that male telomeres shorten faster [94]. A meta-analysis of data from >36,000 subjects revealed a standardized difference in TL between females and males of 0.09 [95]. Based on large-scale LTL data, it was estimated that women are on average 7.4 years younger in biological age than men [84]. One approach to explain this difference is the heterogametic (XY) chromosome setting of male individuals, as some TL maintenance genes are located on the X chromosome, e.g., DKC1 [94]. Another theory is that differences in body size may have an influence on sex-specific TL variation. The larger average body size of males implies that the cells undergo more cellular replication, leading to shorter telomeres [94,96]. Moreover, a higher body mass was found to be associated with stronger repression of telomerase activity in normal somatic tissues [97].

Sex hormones have been shown to influence immune function, development and course of MS as well as telomere maintenance [1,98,99]. The female preponderance in MS is apparent only after puberty, which coincides with a markedly increased production of female sex hormones [100]. Women of reproductive age then have significantly higher levels of estrogen as compared to men. Women with a later age of menarche were found to have a decreased MS risk (odds ratio (OR) = 0.94) [101]. Considerable changes in disease activity have been observed during pregnancy, where the hormone status changes drastically. In the third trimester of pregnancy, relapse rates were found to be reduced by ~70% as compared to before conception [102,103]. Because of this, there is growing interest in using the therapeutic potential of sex hormones in MS [104]. In a randomized phase 2 trial in which female RRMS patients received glatiramer acetate in combination with either oral estriol or placebo for 2 years, a reduced annualized relapse rate (0.25 vs. 0.37) as well as less gray matter atrophy and improved cognition were observed in the estriol group compared with the placebo group [105]. Estrogen confers anti-inflammatory as well as antioxidant properties and is known to promote telomerase expression [99,106]. In a study with postmenopausal women, a high endogenous estrogen exposure, as estimated by the duration of reproductive years, was found to be positively associated with the relative TL in blood cells (age-adjusted *β* = 0.06 for reproductive years vs. T/S ratios) [107]. In men, higher serum levels of estradiol were also shown to correlate with longer LTL (*r* = 0.068) [108].

Testosterone, the primary sex hormone in males, is suggested to have protective effects with regard to MS susceptibility [98]. In a study comparing the occurrence of MS in men with testicular hypofunction (leading to low levels of testosterone) with a control cohort, a ~4.6-fold higher risk of MS was found in the group with testicular hypofunction [109]. Higher testosterone levels were also associated with lower EDSS scores (*ρ* = −0.21) and less cognitive decline [110]. Moreover, in some studies, circulating dihydrotestosterone levels were reported to be positively associated with LTL in men [108,111].

### 3.3. Familial Heritability

Familial recurrence risks of MS have been well studied [35]. Twin studies in MS revealed an overall recurrence risk for monozygotic twins of 18.2% [112]. That means that in comparison to the general population, a monozygotic twin of an MS patient has a roughly 100 times higher risk to develop the disease, which clearly underlines the importance of genetic predisposition in the development of MS. Based on these data, an estimate of overall contributions of genes and environment gave values for genetic heritability (*h*^2^) of 54%, shared familial environment (*c*^2^) of 17% and environment (*e*^2^) of 29% [112]. Siblings with MS are likely to have the same clinical course of MS. However, there is no evidence of intrafamilial concordance of disease severity (e.g., between affected siblings and parent–child pairs) [113].

TL is also a complex genetic trait with two potential sources of heritability: variability in TL of gametes that produce offspring zygotes (direct inheritance) and inherited variation in nontelomeric regions (e.g., single-nucleotide polymorphisms (SNP) in genes that are important for telomere maintenance). TL in parental germ cells impacts TL in offspring cells and contributes to TL heritability despite telomere “reprogramming” during embryonic development [114,115]. A three-generation family-based study reported an estimated LTL heritability *h*^2^ of 0.64 [116]. Significant LTL correlations were found for different types of familial relationships, e.g., between siblings (*r* = 0.22) and between parents and offspring (*r* = 0.15) in age- and sex-adjusted analyses [114]. The heritability of the age-dependent LTL attrition rate was estimated between 24% and 32% [117]. Common SNPs explained approximately 28% of the variance in TL in a study with 3290 European Americans [118]. The individual genetic regions that have been associated with TL are addressed in the following subsection.

### 3.4. Genetic Variants

The major histocompatibility complex (MHC) exerts the largest effect on genetic susceptibility to MS. More precisely, the HLA class II allele HLA-DRB1*15:01 increases the risk of MS (OR = 3.92), whereas the HLA class I allele HLA-A*02:01 has protective effects (OR = 0.67) [119,120]. The global HLA-DRB1 allele frequency distribution might account in part for the higher disease risk in European populations [121]. HLA-DRB1*15:01 has also been reported to be associated with the extent of demyelination in younger MS cases and higher T2 lesion load, resulting in decreased brain volume and cognitive performance [122,123], but overall, no strong contributions of HLA alleles to disease severity or clinical course have been found [120]. Furthermore, more than 200 non-HLA MS risk loci with ORs from 1.06 to 2.06 were identified by genome-wide association studies (GWAS) [124,125]. In a small cohort study, seven non-HLA SNP genotypes were related to the annualized change in EDSS (*β* = 0.11–0.20) and the hazard of relapse [126]. Moreover, a vitamin D receptor gene polymorphism (rs2228570) was found to be associated with reduced disability [127]. However, there is currently no confirmed MS severity locus with genome-wide significance [128,129].

Evidence for a link between TL and HLA genes is weak, but HLA-DRB1*04 alleles were associated with excessive loss of telomeres in CD4^+^ T cells [130]. It has therefore been proposed that HLA-DRB1*04 alleles or genes in linkage disequilibrium (LD) regulate stem cell replication and contribute to senescent and autoreactive T cells. An early GWAS found seven genetic loci to be significantly associated with LTL [131]. Five of those loci harbor genes with known function in telomere biology (e.g., TERT), and the per-allele effect of the different SNPs on LTL ranged from ~57 bp to 117 bp [131]. At two gene loci, the lead SNPs are in LD with MS-associated SNPs in the European population: TERC (rs10936599 vs. rs10936602, *D*′ = 0.841) and RTEL1 (rs2297439 vs. rs6742, *D*′ = 0.492) [125,131,132]. In both cases, the longer TL allele is associated with increased risk of MS. Notably, expression of TERC was found to be significantly higher in monocytes of MS patients relative to healthy subjects [133]. Recently, a prepublished study reported >100 novel genomic loci to be associated with LTL based on data from 472,174 individuals [33]. This study also showed a positive association with MS risk by Mendelian randomization analysis (OR = 1.35 per SD genetically-determined longer LTL) [33]. Last but not least, SNPs in the gene coding for CYP19A1, a neuroprotective enzyme that converts testosterone to estradiol [134], were associated with shorter LTL and lower estradiol levels in serum [108] but not with MS.

## 4. Environmental Factors

### 4.1. Sun Exposure

Sun exposure has received early attention as a risk factor for MS, as it may explain the geographic distribution of the disease, with an increased prevalence of MS at higher latitudes, where the angle of solar radiation is smaller so that less energy reaches the earth’s surface than in equatorial regions [36]. Sunlight provides different forms of radiant energy, including ultraviolet A and ultraviolet B, the latter of which stimulates the production of vitamin D in the skin. Vitamin D-dependent and -independent pathways have been linked to ultraviolet radiation-induced immunosuppression and control of autoimmune diseases [135,136]. A prenatal role of sun exposure in modulating susceptibility to MS has been proposed based on the observation that those born in the spring have an increased risk of MS, possibly because their mothers were pregnant during the winter months [137]. Another observation is that when individuals move before adolescence from an area of high MS prevalence to an area of low prevalence (or vice versa), their MS risk corresponds to that of the region to which they moved [46]. In line with this, a number of studies provided evidence that sun exposure and vitamin D levels in childhood are important modulators of future MS risk [37,138]. For instance, a study by Tremlett et al. showed that living in a high ultraviolet B area and having high summer sun exposure at age 5 to 15 years confers a relative risk (RR) of developing MS of 0.45 [139]. Another study found that the correlation of ultraviolet B exposure with reduced MS prevalence is more pronounced in females (*r* = −0.76) compared to males (*r* = −0.46) [140]. Beneficial effects of sun exposure were also reported for established MS, with higher latitude being associated with worse MS severity scores (MSSS) (*β* = 0.092 per degree in latitude), increased risk of active MS lesions (RR = 1.08) and disability accumulation over time (ΔEDSS) (*β* = 0.067) [141].

Human telomeres are hypersensitive to DNA damage induced by ultraviolet light [142]. In a study by Ma et al., cultured skin fibroblasts were exposed to different doses of ultraviolet A radiation. This revealed a significant inverse relationship between relative TL and ultraviolet A dose (*r* = −0.588) [143]. A small study by Ikeda et al. demonstrated significantly shorter telomeres in epidermal basal cells of sun-exposed skin compared to sun-protected skin [144]. Interestingly, there is growing evidence of crosstalks between senescent stromal cells and skin-resident immune cells [145]. However, the local effects in the skin are quite different from the systemic effects of sunlight. Various studies in the literature focused on the association of short TL with insufficient levels of vitamin D as a possible consequence of deficient sun exposure. We elaborate on the link between TL and vitamin D in Section 5.

### 4.2. Viral Infections

Because of the immune-mediated processes in MS, probability and timing of infections have long been suspected as triggers of disease onset. Of the various pathogens studied, infection with Epstein–Barr virus (EBV) has been most consistently associated with MS. EBV, also known as human herpesvirus 4 (HHV-4), is a widespread human pathogen with ~95% of adults testing seropositive. While EBV infection during childhood is usually subclinical, infection later in life can cause infectious mononucleosis (IM). Studies indicated that ~100% of patients with MS are seropositive for EBV [146,147]. In meta-analyses, anti-EBV nuclear antigen 1 (EBNA1) IgG seropositivity and IM had a summary OR of 4.46 and 2.17, respectively [148]. A particular high risk of MS has been reported for the combination of predisposing HLA alleles and EBV infection (OR = ~15) [37]. In a multiethnic study by Langer-Gould et al., history of IM was associated with a somewhat higher risk of MS in Blacks and Hispanics compared to Whites (OR: 4.43, 3.66 and 2.24, respectively) [149]. With regard to disease severity, it has been described that anti-EBNA1 IgG levels correlate with change in T2 lesion volume (*r*^2^ = 0.26) and EDSS score (*r*^2^ = 0.3) [150]. In line with this, Lünemann et al. found that the immune response against EBNA1 correlated with the number of T2 lesions (*ρ* = 0.24) and EDSS scores after a 1-year follow-up (*ρ* = 0.21) and predicted conversion from CIS to clinically definite MS with a HR of 1.7 [151]. However, another study was unable to show correlations with clinical characteristics, including EDSS and MSSS, but this study did not include MRI outcomes [152].

The relationship between EBV and telomeres appears to be complex. The primary targets of EBV are B cells, where the virus establishes a lifelong latency in the memory compartment. In infected cells, telomere elongation pathways are activated; thus, EBV-carrying B cells with sustained telomerase activity are immortalized [153]. On the other hand, acute EBV infection leads to extensive expansion of EBV-specific CD8^+^ T cells, and telomere shortening in these cells occurs after longer periods of time, possibly because of persistent viral challenge [154]. Interestingly, while EBV infection alone was not found to be related to LTL, a significantly greater LTL attrition over 3 years was seen for individuals coinfected with four different herpesviruses (including EBV) vs. none (*β* = −0.139) [155]. Moreover, higher EBV IgG levels among those who were seropositive were associated with shorter LTL (*β* = −0.023) [155].

The association of HHV-6 type A and B with MS risk has been thoroughly explored as well. Both virus species are ubiquitous, with seroprevalences of >90% in the general adult population. Engdahl et al. reported that the IgG response against HHV-6A is positively associated with MS (OR = 1.55), whereas the IgG response against HHV-6B is protective (OR = 0.74) [156]. Strong immune responses to both HHV-6A and EBV were found to have a significant interaction effect on MS risk [156]. Moreover, HHV-6 IgG titers were associated with the hazard of relapse (with adjusted HR up to 3.10 for the highest stratum) but not with disability scores [157]. A fascinating hallmark of HHV-6 is that it specifically integrates its genome into telomeric regions of chromosomes in infected cells, e.g., CD4^+^ T cells [158]. This provides an elegant mechanism for establishing latent infection in dividing host cells. It has been shown that the telomeres with the integrated viral genome are typically short and unstable [159]. Additionally, seropositivity for HHV-6 was significantly associated with LTL shortening over a follow-up period of 3 years (*β* = −0.033) [155].

HHV-5, also known as cytomegalovirus (CMV), is another common virus, with a seroprevalence of ~65% and ~90% in the European and African populations, respectively [160]. This virus seems to play a protective role in the pathobiology of MS. In a study by Sundqvist et al., infection with CMV was associated with a ~30% decreased risk of developing MS (OR = 0.73) [161]. Another study demonstrated that CMV-seropositive Hispanics, in particular, had a lower risk of MS (OR = 0.49) [149]. With regard to clinical and radiological outcomes, CMV infections have been reported to be favorable or unfavorable, dependent on disease stage. In a study with CIS patients, relapses and conversion to MS over 2 years were more frequently seen in the anti-CMV^+^ group compared to the anti-CMV^−^ group (51% vs. 31% and OR = 2.51, respectively) [162]. On the other hand, in a cohort with established MS, patients positive for antibodies against CMV showed lower relapse rates (*r* = −0.35), and higher titers of anti-CMV IgG were associated with lower T2 lesion load in MRI (*r* = −0.24) [163]. In CMV-seropositive individuals, greater telomere loss in CD4^+^ and CD8^+^ T cells as well as lymphocytes was found compared to that of CMV-seronegative individuals (94 vs. 77 bp/year, 65 vs. 47 bp/year and 61 vs. 45 bp/year, respectively) [164]. This may be explained by changes in the composition of circulating lymphocytes in response to CMV infection, where persistent viral reactivation induces replicative exhaustion and accelerated telomere erosion in CMV-specific T cells [165]. Accordingly, CMV infection was also associated with shorter LTL (*β* = −0.061) after a 3-year follow-up period [155]. Moreover, coinfection with CMV and HHV-6 showed a stronger negative association with LTL than either infection alone (*β* = −0.082) [155].

### 4.3. Other Infections

Besides acute or latent viral infections, bacterial and fungal infections may be implicated in the pathogenesis of MS as well. However, inconclusive findings have been reported so far. For instance, seropositivity for *Helicobacter pylori* (H.p.) was found to be less common in patients with MS than in healthy controls (48.7% vs. 70.9%) [166]. In contrast, a higher prevalence of active H.p. was found in MS patients vs. controls (86% vs. 50%) based on gastric mucosa histology [167]. H.p. IgG seropositivity was significantly associated with short LTL among the elderly (55–75 years, OR = 3.06) but not in the overall population (OR = 1.28) [168]. Recent evidence has suggested that infections with certain combinations of pathogens may decisively contribute to accelerated cellular senescence, possibly beginning early in life. Thus, chronic inflammatory responses secondary to a range of persistent pathogens may have a major impact on markers of cellular aging such as TL [169]. However, the relevance of coinfections and latent pathogen burden in onset and severity of MS remains largely unexplored.

### 4.4. Air Pollution

A significant relation between particulate matter (PM) and prevalence or relapse of MS has been observed in nine studies with rather different methodologies [170,171]. An Italian study showed that in urban areas, where elevated levels of fine particles (PM_2.5_) have been measured on an annual average basis, the prevalence of MS was significantly higher than in villages and the countryside, where PM_2.5_ levels were much lower. A regression analysis underlined that the number of MS patients in a given territorial section was partly related to PM_2.5_ concentrations (*β* = 0.11) [172]. Another study observed that the number of hospital admissions for MS relapses was associated with exposure to inhalable particles (PM_10_) in the preceding week (RR = 1.42 for the fourth vs. the first quartile of PM_10_ exposure) [173]. In contrast, no association of air pollution and MS outcomes was seen in three cohort studies [170].

According to a systematic review, shorter TL has been found to be associated with various air pollutants in the majority of studies conducted so far, although further research is warranted to determine the true effect size of air pollution on TL [174]. Relative TL in buccal cells in adulthood was negatively associated with residential traffic exposure at the birth address (*r* = 0.18) [175]. Moreover, a meta-analysis on early-life traffic-related air pollution indicated that prenatal exposure and exposure during the first year of life to PM_2.5_ and nitrogen dioxide was inversely associated with LTL at age 8 [176]. The underlying mechanisms are presumably linked to oxidative stress and inflammation. Again, this emphasizes the importance of the early-life environment on TL, which may have implications for health outcomes later in life.

## 5. Lifestyle Factors

### 5.1. Dietary Habits

Unlike environmental factors, lifestyle factors are usually modifiable in a more feasible manner. They are therefore of particular interest with regard to the prevention and alleviation of diseases. Obesity in childhood and adolescence is a significant risk factor for MS and is partly a consequence of unhealthy eating behavior and sedentary lifestyle [177]. An increased risk of MS was seen in both females and males with a body mass index (BMI) ≥30 kg/m^2^ at age 20 compared with subjects with a BMI between 18.5 and 21.0 kg/m^2^ (OR = ~2) [178]. In girls, but not boys, obesity was also associated with a significantly increased risk for pediatric MS/CIS (OR up to 3.76) [179]. A causal role of obesity in the etiology of MS was substantiated by a Mendelian randomization study, showing that a genetic predisposition to obesity increases MS risk [180]. Moreover, significant interactions between obesity and HLA alleles with regard to MS susceptibility were found, as subjects with a BMI of >27 kg/m^2^ at age 20 and two risk genotypes (presence of HLA-DRB1*15 and absence of HLA-A*02) had an OR of ~15 compared to nonobese subjects without these genetic risk factors [181]. Interestingly, sex-specific associations between obesity and disease severity were observed: A higher BMI was associated with higher EDSS scores in women (*β* = 0.033) but with lower EDSS scores in men (*β* = −0.053) [182].

A negative correlation of LTL and BMI (*r* = −0.227) and other measures of obesity (e.g., total body fat or waist circumference) was found, especially in those who were younger than 30 years old [183]. A study with children reported that the mean LTL was 23.9% shorter in the obese group than in the nonobese group [184]. Similar findings were reported in other studies: A systematic review identified nine studies with data from children and adolescents, six of which reported an inverse association between obesity and TL [185]. A likely explanation for this dependence is that adipose tissue-derived mediators promote low-grade inflammation and increased systemic oxidative stress, which accelerate telomere shortening [186].

Obesity is generally linked to an unhealthy diet. However, the role of diet in MS has not been comprehensively elucidated so far. A higher consumption of fruits and vegetables typical of the Mediterranean diet was found to be associated with reduced risk of MS [187]. Polyunsaturated fatty acids have been described to have positive effects on MS risk and relapse rate as well [188,189]. Conversely, a higher saturated fat intake and a lower vegetable intake were associated with an elevated hazard to relapse in pediatric MS [190]. Moreover, a study by Farez et al. showed a ~threefold greater chance of relapses and lesion activity in individuals with high sodium intake compared to individuals with low sodium intake [191]. Other studies in turn could not demonstrate effects of salt intake on disease activity [192]. Although there is currently no strong evidence for a direct benefit of dietary interventions on risk and/or course of MS [193], it is important to bear in mind that diet is an important factor influencing the composition of the gut microbiome. Different strains of bacteria were already found to be enriched or depleted in MS cases compared to controls [194]. Further research is needed to clarify whether this imbalance is a cause or a consequence of MS and whether it is related to disease severity.

TL has been associated with dietary patterns as well. Current evidence suggests that adherence to a Mediterranean diet, with consumption of vegetables (e.g., tomatoes as a source of antioxidants and vitamins), fish (rich in omega-3 fatty acids), nuts, seeds and fiber, is associated with longer TL. In contrast, a higher consumption of sugar and dairy products in children and processed meat in adults was associated with shorter TL [195,196,197]. Besides, different strategies for weight loss were reported to delay telomere shortening [198]. However, the possible involvement of gut microbiota in mediating such effects remains to be explored.

### 5.2. Vitamin Supply

Vitamin D can be synthesized in the skin under sunlight exposure. Otherwise, vitamin D status can be improved by eating foods that contain vitamin D (e.g., fatty fish) or by taking supplements. Several studies have demonstrated an association between high vitamin D levels and reduced risk of MS as well as diminished disease activity. More specifically, individuals with 25-hydroxyvitamin D levels >100 nmol/L showed roughly half the likelihood of having MS compared to individuals with <75 nmol/L (OR = 0.49 for Whites and OR = 0.61 for Hispanics) [199]. Moreover, maternal vitamin D deficiency during early pregnancy was found to be associated with an approximately 2-fold increased risk of MS in the offspring (RR = 1.9) [200]. A causal role of vitamin D in MS is also supported by Mendelian randomization studies [201,202]. Additionally, low consumption of fatty fish was associated with increased MS risk (OR = 1.2), although the estimated indirect effect mediated by vitamin D deficiency was small (OR = 1.03) [203]. Notably, patients with higher vitamin D levels were shown to have better clinical and radiological outcomes, with each 25 nmol/L increase in serum 25-hydroxyvitamin D being associated with reduced hazards of relapse (RR = 0.90) and new active lesions in MRI (RR = 0.81) [204]. However, no firm conclusions can be drawn so far about the clinical benefits of vitamin D supplementation in people with MS [205].

Higher 25-hydroxyvitamin D levels in plasma were significantly associated with longer LTL (OR = 1.59 when comparing the highest quartile with the lowest) [206]. Another study reported 0.11 longer relative LTL in participants with sufficient vitamin D status compared to vitamin D-deficient ones [207]. Higher maternal vitamin D concentrations during pregnancy were associated with longer LTL in newborns (*β* = 0.33) [208]. Longer TL was also measured in blood cells of hemodialysis patients with vitamin D treatment compared with untreated ones (9.5 kb vs. 8.4 kb) [209]. However, it has not been definitively resolved whether these associations are related to anti-inflammatory and/or antioxidant effects of vitamin D. Recent studies suggest a crosstalk between vitamin D and the sex hormone estrogen [210].

The role of other vitamins in MS and TL maintenance is less well explored. Patients with MS were found to have relatively low levels of vitamin B-12 (SMD = −0.25) [211], and vitamin B-12 supplement users had ~6% longer LTL than nonusers [212]. Moreover, a significant positive correlation was seen for LTL and intake of vitamin E, which protects against oxidative damage (*r*^2^ = 0.084) [213]. In a study by Guan et al., systemic peroxidation decreased in MS patients treated with vitamin E, but the study duration was too short (3 months) to detect significant effects on disability progression [29].

### 5.3. Drinking Behavior

Studies on the possible relationship of beverages and MS have yielded inconsistent results. In a study by Hedström et al., the risk of developing MS was found to be lower in women and men who reported high alcohol intake as compared with never drinkers (OR = ~0.6) [214]. On the other hand, a significantly increased risk for MS following alcohol abuse and alcohol dependence was seen in men (OR = 1.86 and OR = 1.62, respectively) [215]. High coffee intake (>900 mL daily) was found to be associated with a 30% lower MS risk (OR = 0.7) compared to no coffee consumption [216]. Furthermore, consumption of alcoholic beverages (moderate) and coffee (daily) has been associated with reduced progression of disability in relapsing-onset MS (HR = ~0.6 for the time to sustained EDSS 6) [217]. Another study found that MS patients drinking >3 glasses of red wine per week had, on average, a 0.71 lower MSSS compared to nondrinkers [218]. However, reverse causation and recall bias cannot entirely be ruled out in these kinds of studies.

Chronic alcohol consumption is well known to contribute to premature aging. With regard to cellular aging, alcohol abuse was associated with significantly shorter TL in a recent meta-analysis of six studies (SMD = −0.68) [219]. In contrast, consumption of coffee, which is rich in antioxidants, was related to longer telomeres. Compared with noncoffee drinkers, ORs of having above-median LTL were 1.29 and 1.36 for those drinking 2 to <3 and ≥3 cups of coffee per day, respectively [220]. This is consistent with the results of other studies, as summarized in a review on the impact of beverages and foods on TL by Galiè et al. [197].

### 5.4. Cigarette Smoking

Smoking is a major preventable risk factor for MS. Systematic meta-analyses clearly demonstrated an increased risk of MS due to smoking, especially when comparing ever smokers with never smokers (OR = 1.57) [148,221]. The smoking effect interacts with HLA variants: The combined OR for active smokers having the MS risk allele HLA-DRB1*15:01 but not the protective HLA-A*02 allele was ~14 compared with never smokers without these genetic risk factors [37]. For subjects exposed to passive smoking, the interaction with these HLA genotypes rendered an OR of 7.7 [222]. Smoking also worsens the prognosis in MS [37]. A significant association of active smoking and conversion from RRMS to SPMS was observed (HR = 1.8), with the majority of studies showing evidence of a dose–response relationship [221]. The median time of disability progression (ΔEDSS ≥ 1) of daily smokers and nonsmokers was reported to be 21.3 months and 39.6 months, respectively [223]. Smoking has also been shown to be associated with increased blood–brain barrier disruption, higher lesion volumes and greater atrophy [224]. Therefore, patients with MS should be advised to stop smoking [35,225].

Smoking increases oxidative DNA damage and thus influences telomere shortening. A meta-analysis including 30 studies confirmed significantly shorter TL in peripheral blood cells among ever smokers compared to never smokers (SMD = −0.11) and among current smokers compared to former smokers (SMD = −0.06) [226]. In addition, an inverse trend between pack-years of smoking and mean TL was reported (*β* = −0.01) [226]. Smoking a pack of cigarettes per day for 40 years was shown to provoke accelerated telomere attrition that corresponds to 7.4 years of aging [227]. Intriguingly, estimates for the association between smoking and LTL were larger for women than for men and for Whites than for Hispanic adults [228].

### 5.5. Physical Activity

Physical activity (PA) is an important modifiable lifestyle/behavioral factor that is related to healthy aging. PA prevents obesity and has also been reported to reduce MS risk and severity. Individuals with the highest level of intense early-life PA consistently showed lower risk for developing MS compared to those with the lowest PA level (OR/RR = ~0.7), although it has been debated whether PA may be reduced in response to subclinical MS already before the onset of first neurologic symptoms [229,230,231]. PA was shown to preserve neuromuscular and physical function [232] and to induce neuroprotective effects [233]. In interventional studies with MS patients, aerobic training and other forms of exercise led to improved brain functional connectivity and white matter plasticity as well as a reduced relapse rate (RR = 0.73), as reviewed elsewhere [234]. Thus, regular PA is recommended for patients with MS.

Moderate exercise mediates anti-inflammatory effects and counteracts oxidative stress-related detrimental changes, but acute exercise can generate an excess of free radicals. Accordingly, while several studies have reported that people with higher PA levels have longer TL than those who have a sedentary lifestyle [195,228], too much PA may result in telomere loss [235]. For instance, relative TL measured in saliva were longer in endurance athletes than in controls (1.28 vs. 1.02) but were significantly reduced in response to an ultra-distance endurance trail race (0.86 vs. 1.11), presumably because of oxidative DNA damage [236].

### 5.6. Psychological Stress

Stressful life circumstances might explain the timing of symptom exacerbation for some patients with MS, perhaps by perturbing an already unstable neuroimmunological system [237]. Sickness, accidents or death of a family member and major changes to a romantic or working relationship posed a 14% to 26% increased risk of MS [238]. No elevated risk was seen for individuals who reported physical or sexual abuse during childhood or adolescence in the study by Riise et al. [239]. However, Spitzer et al. reported that patients with MS with histories of physical and/or sexual abuse had a significantly higher relapse rate than patients without severe early-life stress (0.89 vs. 0.62) [240].

A link between different forms of perceived psychological stress (chronic or acute stress in adulthood or traumatic events in childhood) and shortened telomeres has been repeatedly indicated in the literature [241,242]. In a meta-analysis of eight studies, a significant negative correlation between levels of perceived stress and TL in blood cells was determined (*r* = −0.25) [243]. However, any generalization is difficult, as other factors influencing TL, such as diet and exercise, may partly explain these results. It has been argued that stress-related accelerated telomere erosion might be mediated by an anabolic/catabolic hormonal balance, reduced telomerase activity and deficits in antioxidant defense mechanisms [99,244].

### 5.7. Shift Work

Night shift work is a critical health issue because it causes sleep deprivation and circadian rhythm disruption. Hedström et al. investigated the influence of shift work at young adult age on MS risk. In their study, the OR of developing MS was 2.0 for those who had worked shifts for ≥3 years before age 20 years compared with those who had never worked shifts [245]. These findings were confirmed in a later study, with a 1.5-fold increased risk of MS among those who started working shifts before age 20 and a less pronounced association among those who started working shifts at age 20 or later (OR = 1.2) [246]. When considering the intensity of shift work, the risk of MS was found to be increased with an OR of 1.04 for every additional night shift per month [247]. Whether shift work also has an impact on disease severity remains to be clarified, but notably, a small study reported that abnormal melatonin production in patients with MS was related to higher disability and fatigue scores [248].

There is evidence of accelerated telomere shortening in shift workers. In a recent systematic review of the literature, Ledda et al. identified seven studies that underlined that the intensity of shift work is related to modest TL shortening [249]. For instance, a significant decrease in LTL was seen in nurses having worked in night shifts for ≥12 years (*β* = −0.07) [250], and salivary TL was shortest among those who worked >4 consecutive night shifts for ≥5 years [251].

## 6. Concluding Remarks

In the last years, research on telomeres and biological aging has gained increasing interest. Evidence is accumulating that LTL provides an indication of immunocompetence. For instance, higher risks of infection (HR = 1.05) and infection-related death (HR = 1.10) per SD shorter LTL were determined in a large study with 75,309 individuals [252]. Meanwhile, shortened telomeres have been reported in diverse autoimmune and neurological diseases [25,26,253]. There have to date been six relatively small studies that investigated TL in up to 138 patients with MS in comparison to controls [32]. Four of these studies found shorter TL in the peripheral blood in at least one MS subtype [28,29,30,31], and one study concluded that telomere shortening was accelerated in mesenchymal stromal cells of MS patients [254]. In a cohort study by Krysko et al., shorter LTL were associated, independently of age, with greater disability, lower brain volume and an increased relapse rate [255]. Additionally, our group found that RRMS patients with relatively short LTL were at much higher risk to convert to SPMS [31]. Here, we reviewed the current literature for genetic, environmental and lifestyle factors that have been linked to TL and that may contribute to the risk of developing MS and possibly the heterogeneity in the clinical course of patients with MS.

We found various shared determinants of TL and MS, suggesting that the mechanisms underlying the disease are connected to premature cellular aging and senescence. Multiple lifestyle factors were found to correlate with reduced TL and higher MS risk, with the most robust findings seen for obesity, lack of physical activity, smoking and vitamin D deficiency. The accelerated telomere attrition in individuals with these risk factors is presumably mediated by increased inflammation and oxidative damage. Childhood and adolescence is the period of life where some factors have their greatest impacts. The decline of LTL with age is generally more rapid in the first years of life (before puberty) and is related to a high leukocyte turnover [256]. Thus, LTL dynamics in the first 20 years of life have a dominant effect on LTL for the remaining lifespan [117]. On the other hand, early-life factors appear to largely determine susceptibility to MS and, to some extent, the course of disease later in life. However, the importance of timing of risk factors is difficult to study, and there is an overlap of different influences. For instance, it is complicated to assess the impact of vitamin D status, which depends on seasonal variations in sun exposure at different latitudes, time in the sun, amount and pigmentation of exposed skin, diet and supplement use. Thus, there may be evidence from different directions, including genetic insights from Mendelian randomization studies, which in this case have supported a causal role of vitamin D in the pathogenesis of MS [201,202]. A few factors so far have been associated either with MS risk only (e.g., exposure to organic solvents [257,258]) or with TL only (e.g., sleep duration [259,260,261]). These factors were not included in our inventory, but certainly deserve further investigation in future studies.

It is important to emphasize that the associations of genetic, environmental and lifestyle factors with TL and MS do not generally prove causal relations. Potential biases may result from confounding and reverse causation. A huge number of factors constantly influence each other, which provokes chaotic dynamics, similar to those outlined by the n-body problem in celestial mechanics [262]. Notably, the course of the disease may obey other chaotic rules than those in effect before the onset of MS. Therefore, we need more accurate knowledge about the “gravitational forces”, here to be understood as the interactions of genetic and nongenetic factors. High MS risk ORs of ~15 have already been established for the presence of the HLA-DRB1*15:01 allele and the absence of the HLA-A*02:01 allele in combination with either smoking, EBV infection or adolescent obesity [37]. In a recent publication by Hedström et al., a three-way interaction of overweight status (BMI ≥ 25 kg/m²) at age 20, HLA-DRB1*15:01 and history of IM led to an OR as high as 22.2 [263]. Interactions have also been studied with regard to TL. For instance, in analyses stratified by sex and race/ethnicity, the association of heavy versus no/minimal smoking with LTL was found to be particularly strong among Black women [228]. Further research is needed to elucidate additional gene–environment interactions that play a role in MS and telomere biology.

There are several limitations that should be considered in the interpretation of the data. Most associations have been demonstrated for people of European ancestry, and it remains somewhat unclear whether they are similarly present in other populations. In the field of MS, most of the evidence is based on patients with RRMS, as patients with PPMS are typically underrepresented in studies. Recent reports have shown that healthcare use is higher in the 5 years before MS symptom onset (RR from 1.2 to 1.9) [264]. This so-called “MS prodrome” is of critical concern for studies on MS risk factors and therefore needs to be better understood. Studies on potential disease modifiers are often limited by small sample size and huge heterogeneity in the assessment of disease progression. In fact, the establishment of more quantitative and objective disease outcome measures remains a key priority in MS research [265]. There is also still a lack of large-scale prospective studies, for instance, to evaluate the individual effects of dietary patterns and components as well as physical activity and exercise interventions on TL and MS outcomes. Moreover, TL was measured mostly by TRF or PCR analysis in whole blood and rarely in other tissues. More precise insights could be obtained by using advanced techniques to measure absolute TL and TL distributions in specific cell populations. LTL is a predictor of global and regional brain volumes [266], and a shared genetic relationship between LTL and brain morphological traits has been revealed [267]. However, further studies are needed to determine how TL and anatomic structural changes in the CNS are related to cognitive function and neurological disease.

The immune system is involved in basically all factors discussed in our review. In MS and other chronic immune-mediated diseases, a few (autoreactive) cells are believed to control a vast population of nonspecific cells. Dissections of MS genetic signals refined the driver subsets to CD4^+^ Th17 cells, memory B cells and microglia [125,268]. Therefore, it would be interesting to explore the telomere dynamics in these cell types as well as the functional implications. Microglia, the primary antigen-presenting cells in the brain, have a significant mitotic potential and are thus susceptible to telomere shortening. Senescence-driven impairments of microglia responses have been suggested to play essential roles during onset and progression of neurodegenerative diseases [269]. Senescent cells accumulate during aging, and their senescence-associated secretory phenotype (SASP) has been proposed as the main contributor to inflamm-aging [67,270]. However, the regulation of senescence-related pathways and telomere maintenance genes in MS is currently not well understood. Cytokines and proteases produced by senescent cells may promote MS progression, e.g., by altering the integrity of the blood–brain barrier [63]. While Hug et al. found no increased telomerase activity in CD4^+^ and CD8^+^ T cells from MS patients [271], Thewissen et al. measured significantly reduced TERT mRNA levels in stimulated blood cells of patients with MS as compared to healthy controls [272]. This warrants further investigation.

Knowledge of modifiable risk factors can help mitigate the risk of developing MS, alleviate disease symptoms and prevent premature telomere loss. General health recommendations can be followed to reduce the risk of MS, particularly in at-risk family members: avoidance of exposure to cigarette smoke, regular sun exposure and prevention of childhood obesity by promoting a Mediterranean diet and exercise. The same behaviors may be recommended for patients with established MS, with reasonable confidence for their likely benefits to overall health, even if MS-specific benefits are not yet definitely clear [192]. This should be kept in mind when counseling patients and their relatives in clinical practice.

The effect of disease-modifying treatments for MS on immunosenescence and TL of specific immune cells is not yet clear. However, most drugs used in MS are directly or indirectly related to the defense against OS [273]. Supplementation with vitamins and other antioxidants, which have the potential to reduce OS biomarker levels and the rate of TL shortening, may provide additional clinical benefits at least in a subgroup of patients with MS. The modulation of telomerase activity is a novel therapeutic option that is currently being tested for other diseases, but there is significant potential for undesirable side effects, and therefore, optimized delivery systems are required to reach the specific target cells [274]. Another approach might be to eliminate senescent cells and the accompanying SASP by so-called senolytics. While senolytics have proved beneficial in in vitro and animal models [275], it remains to be shown whether they are safe in humans and perhaps effective in treating MS.

The ever-increasing volume of digitized health information will help to learn more and more about genetic, environmental and lifestyle/behavioral factors that influence both aging and MS. This may lead to better estimates of the weight of each factor on overall disease risk, thereby enabling more refined personalized risk models. The further elucidation of interaction effects of risk factor combinations should be intensified in interdisciplinary research efforts. More robust studies are needed to determine how a modified individual lifestyle can influence cell type-specific telomere dynamics while leading to more favorable disease outcomes and improved quality of life. Novel insights into the molecular mechanisms underlying development and progression of MS are likely to be gained through a better understanding of cellular biological aging phenomena. The assessment of TL and of other biomarkers of DNA damage and telomere dysfunction might be also considered in the design of large-scale observational studies and clinical trials in order to correlate the data with the age-related symptomatology and course of individual MS patients. Finally, targeting senescent cells may be a promising complementary approach in the future therapeutic management of MS.

## Figures and Tables

**Table 1:** Factors associated with risk and possibly severity of multiple sclerosis as well as telomere length.

	MS Risk		MS Severity		TL	
Factor	Effect	Evidence	Effect	Evidence	Effect	Evidence
**Genetics**						
Ancestry (European)	➚	***	➘	*	➘	***
Sex (female)	➚	***	•	***	➚	***
Estrogen in women	➚	*	➘	**	➚	*
Testosterone in men	➘	*	➘	*	➚	*
Familial heritability	•	***	−	−	•	***
HLA alleles	•	***	•	*	•	*
SNPs	•	**	−	−	•	**
**Environment**						
Sun exposure	➘	**	➘	*	•	*
Epstein–Barr virus	➚	***	➚	**	•	*
HHV-6A	➚	*	➚	*	➘	**
CMV	➘	*	•	*	➘	**
Bacterial infections	•	*	−	−	➘	*
Air pollution	➚	*	➚	*	➘	**
**Lifestyle**						
Obesity	➚	***	•	*	➘	***
Mediterranean diet	➘	*	➘	*	➚	**
Vitamin D sufficiency	➘	**	➘	*	➚	**
Other vitamins	➘	*	−	−	➚	*
Alcohol	•	*	➘	*	➘	*
Coffee	➘	*	−	−	➚	*
Smoking	➚	***	➚	***	➘	***
Physical activity	➘	**	➘	**	•	**
Psychological stress	➚	*	➚	*	➘	**
Night work	➚	**	−	−	➘	*

The genetic, environmental and lifestyle factors are given in the order in which they are mentioned in the text. The reference group is given in parentheses for ancestry and sex. The upward arrows represent greater risk of MS, worse disease phenotype (with more rapid accumulation of disability and/or more relapses and brain lesions/atrophy) or longer telomeres. The red color indicates worse outcomes, reflected by higher disease risk, more severe disease activity/progression or accelerated telomere shortening, if having or being exposed to a particular risk factor. The blue dot indicates that the association is (or may be) context dependent. For instance, the effect may depend on the specific allele of a genetic variant (e.g., SNP) or on the degree of exposure (e.g., alcohol consumption). The level of evidence, according to the authors’ personal assessment, is given by asterisks, with *** indicating a particularly strong association and a rather consistent body of research (i.e., the findings of large studies were replicated and/or supported by different approaches). With regard to severity of MS, smoking is the only established modifiable factor with the highest level of evidence, whereas for some other factors, there is no conclusive evidence so far (−). For most factors, there is an inverse relationship to MS risk and TL. Effect sizes and references to the literature are given in the text. CMV: cytomegalovirus, HHV: human herpesvirus, HLA: human leukocyte antigen, MS: multiple sclerosis, SNP: single-nucleotide polymorphism, TL: telomere length.

## Glossary

**Alternative lengthening of telomeres (ALT):** a telomerase-independent pathway of TL elongation that is based on the activation of a homologous recombination DNA repair mechanism and the utilization of other telomeric DNA as a template.

**Biological aging:** the decline in physical and mental functions over time due to gradually accumulating damage to various cells and tissues in the body. The biological age of an individual (which may be estimated by TL or DNA methylation analysis) can differ substantially from their chronological age (the number of years the person has been alive).

**Cellular senescence:** a state of irreversible cell cycle arrest that can be initiated by short dysfunctional telomeres and a wide variety of stress-inducing factors. Senescent cells secrete proinflammatory cytokines, growth factors and proteases, a phenomenon called senescence-associated secretory phenotype (SASP).

**End-replication problem:** the ends of linear chromosomes cannot be replicated completely during DNA synthesis. Each round of DNA replication leaves 50–100 bp DNA unreplicated at the 3′ end, thereby leading to telomere attrition during cell proliferation.

**Genome-wide association study (GWAS):** examination of many genetic variants (usually SNPs) across the entire genome in many people. Genetic variants found at an increased frequency in people with a particular condition compared with people without the condition are said to be associated with the condition. This can point to genetic differences that correlate with and perhaps play a causative role in a disease (such as MS) or trait (such as TL) of interest.

**Hayflick limit:** the inability of cells to divide (replicate) indefinitely. Cell division ceases once telomeres shorten to a critical length. Fetal human cells have a uniform limit of ~50 divisions.

**Hazard ratio (HR):** an estimate of the ratio of the hazard rates of two groups to be compared. The hazard rate is the probability that if the event in question has not already occurred, it will occur during a follow-up time interval.

**Heritability:** the proportion of variance in a phenotype that can be attributed to variation of genetic factors among individuals in a given population as opposed to variation of environmental factors.

**Immunosenescence:** the weakening of the immune system with increasing age. Because of age-related changes in immune cell composition, there is a decrease in the ability to mount effective antibody and cellular responses against infections and to vaccinations.

**Inflamm-aging:** a chronic low-grade inflammation in aging individuals, possibly as a result of persistent exposure to infectious agents. The overproduction of proinflammatory molecules is believed to accelerate biological aging and to worsen disease prognosis.

**Mendelian randomization:** the use of genetic variants that typically serve as a proxy for a nongenetic (modifiable) risk factor in order to make causal inferences about the effect of an exposure on disease and health-related outcomes. The approach is inspired by Mendel’s second law.

**Odds ratio (OR):** measure of association that compares the chance of an event occurring in one group to the chance of it occurring in another group. In the context of disease, the ratio of the odds of developing the disease between individuals exposed and unexposed to a factor (e.g., genotype or environmental exposure).

**Relative risk (RR):** the ratio of the probability of an outcome in an exposed group to the probability of an outcome in an unexposed group. Unlike the OR, it is calculated from the cumulative incidence obtained from prospective data.

**Senolytics:** a class of drugs under basic research because of their capacity to selectively induce death of senescent cells and thereby prevent or alleviate age-related pathologies.

**Single-nucleotide polymorphism (SNP):** the most common type of genetic variation, in which a single base (A, T, C or G) at a specific chromosomal location differs among individuals in the population. SNPs usually consist of two forms (alleles).

**Standardized mean difference (SMD):** the difference between two means divided by an estimate of the within-group standard deviation. It is commonly used as a summary statistic in me-ta-analysis when the studies under review did not use the exact same outcome measure.

**Telomerase:** the ribonucleoprotein enzyme complex that adds telomeric DNA repeats to the ends of chromosomes. Telomerase activity promotes cellular immortality. However, it is absent or expressed at very low levels in normal human somatic cells.

## Abbreviations

8-OHdG	8-hydroxy-desoxyguanosine
ALT	alternative lengthening of telomeres
BMI	body mass index
bp	base pair
CHI3L1	chitinase 3-like protein 1
CIS	clinically isolated syndrome
CMV	cytomegalovirus
CNS	central nervous system
CRP	C-reactive protein
EBNA1	Epstein–Barr virus nuclear antigen 1
EBV	Epstein–Barr virus
EDSS	Expanded Disability Status Scale
GWAS	genome-wide association study
HHV	human herpesvirus
HLA	human leukocyte antigen
H.p.	*Helicobacter pylori*
HR	hazard ratio
IgG	immunoglobulin G
IM	infectious mononucleosis
kb	kilobase
LD	linkage disequilibrium
LTL	leukocyte telomere length
MHC	major histocompatibility complex
MRI	magnetic resonance imaging
MS	multiple sclerosis
MSSS	Multiple Sclerosis Severity Score
OR	odds ratio
OS	oxidative stress
PA	physical activity
PCR	polymerase chain reaction
PM	particulate matter
PPMS	primary progressive multiple sclerosis
Q-FISH	quantitative fluorescence in situ hybridization
RNA	ribonucleic acid
RR	relative risk
RRMS	relapsing-remitting multiple sclerosis
S	single-copy gene signal
SAA	serum amyloid A
SASP	senescence-associated secretory phenotype
SD	standard deviation
SMD	standardized mean difference
SNP	single-nucleotide polymorphism
SPMS	secondary progressive multiple sclerosis
T	telomere signal
TERC	telomerase RNA component
TERT	telomerase reverse transcriptase
TeSLA	telomere shortest length assay
TL	telomere length
TRF	terminal restriction fragment
U-STELA	universal single telomere length analysis
